# Simultaneous LC–MS/MS method for the quantitation of Azithromycin, Hydroxychloroquine and its metabolites in SARS-CoV-2(−/ +) populations using dried blood spots

**DOI:** 10.1038/s41598-023-43185-9

**Published:** 2023-09-30

**Authors:** Yashpal S. Chhonker, Wafaa N. Aldhafiri, Dhruvkumar Soni, Neerja Trivedi, Claire Steinbronn, Christine Johnson, Helen C. Stankiewicz Karita, Michael K. Paasche-Orlow, Ruanne Barnabas, Samuel L. Arnold, Daryl J. Murry

**Affiliations:** 1 Clinical Pharmacology Laboratory, Department of Pharmacy Practice and Science, College of Pharmacy, University of Nebraska Medical Center, Omaha, NE 68198-6025 USA; 2 Department of Pharmaceutics, University of Washington, Seattle, WA 98195 USA; 3 Department of Medicine, University of Washington, Seattle, WA 98195 USA; 4 Department of Medicine, Tufts Medical Center, Boston, MA 02111 USA; 5 Division of Infectious Diseases, Massachusetts General Hospital, Boston, MA 02114 USA; 6 Harvard Medical School, Boston, MA 02115 USA; 7 Fred and Pamela Buffett Cancer Center, University of Nebraska Medical Center, Omaha, NE 68198 USA

**Keywords:** Bioanalytical chemistry, High-throughput screening, Drug safety

## Abstract

Severe acute respiratory syndrome coronavirus 2 (SARS-CoV-2) led to a global pandemic of coronavirus disease 2019 (COVID-19). Early in the pandemic, efforts were made to test the SARS-CoV-2 antiviral efficacy of repurposed medications that were already approved and available for other indications, including hydroxychloroquine (HCQ) and azithromycin (AZI). To reduce the risk of SARS-CoV-2 exposure for clinical-trial study participants and to conform with lockdowns and social distancing guidelines, biospecimen collection for HCQ and AZI included at-home dried blood spot (DBS) collection rather than standard venipuncture by trained clinicians. In this study, we developed and validated the first sensitive and selective simultaneous LC–MS/MS method to accurately quantitate the concentration of HCQ, HCQ metabolites (Desethylchloroquine [DCQ], Bisdesethylchloroquine [BDCQ], Monodesethylhydroxychloroquine [DHCQ]) and AZI extracted from DBS. The validated method was successfully applied for the quantification of over 2000 DBS specimens to evaluate the pharmacokinetic profile of AZI, HQC, and its metabolites. This new method has a small sample volume requirement (~ 10 µL), results in high sensitivity (1 ng/mL), and would facilitate remotely conducted therapeutic drug monitoring.

## Introduction

A novel coronavirus, severe acute respiratory syndrome coronavirus 2 (SARS-CoV-2), was identified in January of 2020 as the primary cause of a viral pulmonary infection outbreak in Wuhan, China. The disease was later named COVID-19 and spread globally, causing a pandemic that was declared a public health emergency of international concern by the World Health Organization (WHO)^1–3^. Because antiviral therapies and vaccines were unavailable at the start of the pandemic, efforts were made to test the SARS-CoV-2 antiviral efficacy of repurposed medications that were already approved and available for other indications^4^. Two such medications were hydroxychloroquine (HCQ) and azithromycin (AZI).

HCQ is a widely used anti-malarial, anti-inflammatory, and immunomodulatory treatment^1,5^. It was first synthesized in 1946 as a soluble and less toxic hydroxyl analog derivative of chloroquine (CQ)^1,6–9^. HCQ is metabolized into three major metabolites desethylchloroquine (DCQ), bisdesethylchloroquine (BDCQ), and monodesethylhydroxychloroquine (Cletoquine; DHCQ), yet the activity and toxicity of HCQ metabolites are poorly understood^10^.

Beginning of the twenty-first century, the scientific world was interested in repurposing HCQ in the treatment of other infectious diseases^10–13^. Initially as an antibacterial agent against *Coxiella burnetii* infections responsible for causing chronic Q fever^14,15^, and later as an antibacterial against hospital-acquired *Staphylococcus aureus*^11–13,16,17^ and an antifungal against *Histoplasma capsulatum*^11,17,18^. Repurposing of HCQ as an antiviral agent has been explored in the context of multiple pandemics, including human immunodeficiency virus (HIV)^19,20^, Ebola^13,21^, Zika^22,23^, and SARS-CoV outbreak in (2002 and 2003)^22–26^. Even though antiviral efficacy has not been confirmed against any of these viruses, HCQ emerged as a potential SARS-CoV-2 antiviral therapy due to the similarity between SARS-CoV outbreak and SARS-CoV-2. HCQ, alone and in combination with AZI, appeared to be associated with reduced viral shedding in vitro and in small observational studies early in the pandemic, but were later shown not to effect SARS-CoV-2 acquisition, shedding, or disease in rigorous randomized controlled trials^27–30^.

To conduct rigorous clinical trials on HCQ, HCQ metabolites, and AZI in the context of the SARS-CoV-2 pandemic, researchers relied on at-home biospecimen collection by participants, including self-collection of dried blood spots (DBS) instead of standard venipuncture by a phlebotomist. This approach reduced the risk of SARS-CoV-2 exposure for study participants and clinical staff and was aligned with COVID-19 home quarantine guidelines that were issued in numerous US jurisdictions during the first year of the pandemic. Additional advantages of DBS sample collection include that it is less invasive, allow patient sample self-collection, it requires only a small blood volume, and that DBS samples that don’t contain infectious agents can be stored at ambient temperature, reducing logistical barriers to specimen storage and transport^27,28^. A barrier in DBS application at the onset of the COVID-19 pandemic was the lack of validated methods existing to assess the concentration of HCQ, HCQ metabolites, and AZI from DBS.

We previously reported the development and validation of a selective and sensitive liquid chromatography with tandem mass spectrometry (LC–MS/MS) method for the quantitation of HQC and its metabolites, along with structural information, using 100 µL of mouse blood and tissue samples^29^. A LC–MS/MS method for the quantification of HCQ, its metabolites (DHQC and BDCQ), and AZI in human plasma has recently been reported^30^. However, these earlier methods require a large sample volume that can only be accessed through a clinic visit.

In the present research, we developed and validated a sensitive LC–MS/MS method for simultaneous quantitation of AZI, HCQ, and its major metabolites (DCQ, BDCQ, and DHCQ) from DBS. We applied the method to DBS samples collected from clinical trials investigating HCQ for COVID-19 pandemic post-exposure prophylaxis (ClinicalTrials.gov: NCT04328961) and early treatment for COVID-19 (ClinicalTrials.gov: NCT04354428)^31,32^, where HCQ and AZI drug combinations were administered. This method will facilitate future use of DBS for assessing blood concentrations of AZI, HCQ, and its metabolites for future remotely conducted therapeutic drug monitoring involving these drugs. It also provides the framework for home sampling while decreasing the burden on clinical sites during a pandemic.

## Materials and methods

### Chemicals and materials

AZI, HCQ, HCQ metabolites (DCQ, BDCQ, DHCQ), and internal standards (IS) HCQ-d4, BDCQ-d4, and AZI-d3 of pharmaceutical grade were obtained from Sigma-Aldrich (St Louis, MO). HPLC grade methanol (MeOH), formic acid (FA), Whatman 903 protein saver cards and acetonitrile (ACN) were purchased from Fisher Scientific (Fair Lawn, NJ). Ultrapure water was produced using a Barnstead GenPure water purification system (ThermoScientific, Waltham, MA). A Resprep VM-96 Vacuum Manifold was used for sample processing (Restek Bellefonte, PA). Human blood was obtained from the ZenBio (Durham, NC) containing acid citrate dextrose (ACD) as an anticoagulant and transported under refrigerated conditions inside an ice pack . All other reagents used were of analytical grade or higher and procured from standard chemical suppliers. Institutional approval was obtained for receiving DBS samples from subjects with potential COVID-19 infections (UNMC IBC# 20-04-025-BL1). During the handling of all reagents, appropriate personal protective equipment (PPE) such as gloves, goggles, and face shields were used. DBS samples were also handled with care to minimize the risk of exposure using engineering controls such as hoods. Working solutions that contained hazardous materials such as DMSO were properly labeled with hazard statements.

### Liquid chromatographic and mass spectrometric (LC–MS/MS) conditions

An LC–MS/MS 8060 system (Shimadzu Scientific Instruments, Columbia, MD, USA) equipped with a dual ion source (DUIS), operated in positive electrospray ionization mode, was used to perform mass spectrometric detection. A Shimadzu Nexera UPLC system equipped with two pumps (LC-30 AD), a column oven (CTO-30AS) and an auto-sampler (SIL-30AC) was used for sample injection and cooling (4 °C). LabSolutions LC–MS software Version 5.99 SP2 (Shimadzu Scientific, Inc., Columbia, MD, USA) was used for instrument control, data acquisition and quantitation. MS parameters, such as temperature, voltage, gas pressure, etc. were optimized by auto method optimization through a product ion search using a 1.0 µg/mL solution in MeOH. Instrument condition and detection parameters are summarized in (Table 1).

**Table 1:** Summary of liquid chromatography with tandem mass spectrometry (LC–MS/MS) conditions.

Parameter	Condition	
LC–MS/MS model	LC–MS/MS 8060 system (Shimadzu Scientific Instruments, Columbia, MD) equipped with a dual ion source (DUIS)	
LC–MS/MS software	LabSolutions LCMS software Version 5.99 SP2 (Shimadzu Scientific, Inc., Columbia, MD)	
LC–MS/MS ionization mode	Positive electrospray ionization (ESI +) mode	
MS parameters	Nebulizing gas:	2.0 L/minute
	Drying gas flow:	10.0 L/minute
	Heating gas flow:	10.0 L/minute
	Interface temp:	300 °C
	DL Temp:	250 °C
	Block heater temp:	400 °C
Analytical LC column	AQUASIL C18 (5 μm, 50 × 4.6 mm, part number: 77505–054630, ThermoScientific, Waltham, MA) protected with a AQUASIL C18 guard column (ThermoScientific, Waltham, MA)	
Column pressure	1000 psi	
Column Temperature	40 °C	
Mobile phase A	Water with 0.2% v/v formic acid	
Mobile phase B	Methanol with 0.1% v/v formic acid	
Flow	0.5 mL/minute	
Gradient elution	Time (minute)	%B
	0.01	Inject
	0.1	20
	2	20
	6	70
	7.5	90
	7.6	20
	8.1	Stop
Total run time	8.1 min	
Injection volume	10 µL	

The multiple reaction monitoring (MRM) transitions for each analyte and IS, as well as their respective optimum MS parameters, such as voltage potential (Q1, Q3), and collision energy (CE), are shown in Table 2.

**Table 2:** Summary of MS/MS parameters: precursor ion, fragment ions, voltage potential (Q1), collision energy (CE) and voltage potential (Q3), retention time, and linearity for analytes.

Analyte	Type	MRM	Q1 (V)	CE (V)	Q3 (V)	Retention Time	Linearity (ng/mL)	Sensitivity on column (pg)
HCQ	Target	336.10 > 247.20	− 11	− 21	− 18	4.8	1–2000	0.11–217.40
DCQ	Target	292.10 > 114.20	− 10	− 21	− 12	4.7	1–2000	0.11–217.40
BDCQ	Target	264.00 > 179.00	− 11	− 22	− 13	4.3	1–2000	0.11–217.40
DHCQ	Target	308.15 > 179.00	− 19	− 25	− 13	4.3	1–2000	0.11–217.40
AZI	Target	749.50 > 591.45	− 40	− 29	− 22	6.6	1–2000	0.11–217.40
HCQ-d4*	IS	340.10 > 251.20	− 12	− 22	− 18	4.8	NA	NA
BDCQ-d4*	IS	268.10 > 179.00	− 29	− 23	− 30	4.3	NA	NA
AZI-d3*	IS	754.50 > 596.45	− 40	− 31	− 22	6.6	NA	NA

(*HCQ*) Hydroxychloroquine, (*DCQ*) desethylchloroquine, (*BDCQ*) bisdesethylchloroquine, (*DHCQ*) monodesethylhydroxychloroquine, (*AZI*) azithromycin.

*HCQ-d4 was used as the IS for HCQ. *BDCQ-d4 was used as IS for DCQ, BDCQ and DHCQ. *AZI-d3 was used as the IS for AZI.

### Preparation of stock, calibration standards and quality control samples

Stock solutions of 1 mg/mL of all analytes were prepared as follows: 1 mg of HCQ was dissolved in 500 µL water then 500 µL MeOH . 1 mg of AZI, DCQ, and BDCQ were directly dissolved in 1 mL MeOH. DHCQ was initially dissolved in 200 µL DMSO then 800 µL MeOH. Considering the potential enhanced toxicity of the DHCQ stock solution prepared in the DMSO: MeOH (2:8, v:v) solvent system, appropriate personal protective equipment (PPE) such as gloves, lab coat, safety goggles, and respiratory protection were used. The solution was prepared in a well-ventilated area or under a fume hood and stored in a hazardous labeled, tightly sealed container.

The IS HCQ-d4, BDCQ-d4, and AZI-d3 were prepared as 1 mg/mL solutions in MeOH. The IS were selected as the deuterated analogues of our compounds of interest.

Two independent stock solutions (1 mg/mL) were prepared for calibration standards (CCs) and quality control (QCs). The stock solutions were diluted with MeOH to make different mix working standards (MWS:0.04, 0.08, 0.2, 0.4, 0.8, 4, 8, 40, 70, 80, 0.04, 0.12, 20, and 60 µg/mL).The CCs and QCs were prepared by spiking 2.5 µL of MWS solution into 97.5 µL of human blood to obtain a concentration range of 1–2000 ng/mL (1, 2, 5, 10, 50, 100, 500, 1000, 1700 and 2000 ng/mL).

The QCs comprised of four different concentration levels, including the lower limit of quantification (LLOQ; 1 ng/mL), the low QC (LQC; 3 ng/mL,), middle QC (MQC; 500 ng/mL), and high QC (HQC; 1500 ng/mL). QCs were prepared separately in six replicates, independent of CCs stocks. Further, these CCs and QCs blood samples were used to generate DBS controls as per Section “Preparation of DBS and extraction method”. All the main stocks were transferred to a 2 mL vial and stored at − 80 °C. Intermediate stocks, MWS stocks, and QCs stock solutions were kept at -20 °C until use or prepared fresh when needed.

### Blood and DBS sample preparation

#### Hematocrit effect adjustment

Hematocrit (Hct) has a direct effect on the accuracy and precision of the qualitative analysis of DBS^27,28,33^. To evaluate Hct effect on the calibration standards, blood with different Hct was prepared. In a micro-centrifuge tube, 300 µL of blood was centrifuged at 4000 × *g* for 10 min at 4 °C to separate the plasma. The separated plasma was removed or added to the separated hematocytes to make a range of Hct values (0.2, 0.4, and 0.6) for both LQC and HQC.

#### Preparation of DBS and extraction method

Upon receiving the control blood, we promptly created the dried blood spot (DBS) samples and stored them in − 80 °C. The DBS samples were utilized within 7 days of storage. Samples were prepared by spiking 2.5 µL of the appropriate MWS into 97.5 µL blank matrix (40 × diluted). To avoid changing the blood sample nature, we limited the volume of the analyte solution to < 5% of the blood matrix volume. The spiked blood samples were left for 3 min to reach red blood cell (RBC)/plasma equilibrium at a 37 °C temperature to mimic *in-vivo* samples' RBC/plasma analyte distribution. After equilibration, 40 µL of the spiked blood was spotted on Whatman 903 filter paper and left to dry for 3 h at room temperature. A BSD Duet device (Model No. BSD600 / DUET, BSD Robototics Brendale, Australia) was used to punch two 3 mm samples from each CC, QCs and human DBS card. The punches were collected in 96 well plates and diluted with 100 µL 1% FA water and 10 µL of mixed IS solution (0.2 µg/mL). The 96 plates were then vortexed for 5 min on a Mixmate (Eppendrof, CT, USA) at 1200 rpm and then sonicated for 10 min. Ice chilled MeOH (350 µL) was added to each well of the 96 plate and vortexed for 5 min on a Mixmate at 1200 rpm, then transferred to Phree 96 well plate (Phenomenex Inc, Torrance CA.) and centrifuged at 3000 rpm for 10 min. After centrifugation, 70 µL of the eluate was collected and diluted with 70 µL of 0.1% FA water in 96 round bottom plates and 10 µL of the resultant mixture was injected into the LC–MS/MS system.

### Assay validation

The developed LC–MS/MS method was fully validated in accordance with the 2018 Food and Drug Administration (FDA) guidelines and regulation for Bioanalytical Method Validation^34^ with respect to selectivity, specificity, linearity, accuracy, precision, matrix effect, stability, and dilution response.

#### Selectivity, specificity, and sensitivity

In order to assess the specificity and selectivity of our assay, we analyzed six individual donor lots of blood samples for any potential interference at the analyte retention time. We also tested hematocrit effect (Hct) on QCs. The sensitivity determination involved calculating the signal-to-noise ratio (S/N) of the analyte response in the calibration standards. The S/N ratio was determined by comparing the baseline noise at the retention time of the analyte with that of the lower limit of detection (LLOD) peak response. We required the S/N ratio to be greater than three for the LLOD and greater than ten for the LLOQ.

#### Linearity and calibration curve

The calibration curve was established by plotting the analyte/IS peak area ratio (the y-axis) versus the nominal concentration of each standard (the *x*-axis). The calibration curve was generated using a weighted least-squares linear regression (1/x^2^). Each standard curve consisted of a blank sample, a zero sample (blank + IS), and 10 non-zero concentrations consisting of ten CCs. At least 75% of the standards were required to be within ± 15% of the nominal concentration except at LLOQ, which should not deviate by more than 20% for acceptance of the generated calibration curve. A minimum of four out of six QC samples were required to be within ± 15% of their respective nominal values to accept the generated calibration curve.

#### System suitability and carry-over effect

System suitability was monitored before validation. At the beginning of the analytical run five system suitability HQC samples were injected into the system to assess the retention time, peak area and carry over. The % CV of analyte/IS peak area ratio obtained from the five system suitability samples should be less than 6%. Carryover was assessed by measuring the analyte response in a blank sample following the injection of the HQC. Carry-over was required to be less than 20% of the response of a processed LLOQ sample.

#### Accuracy and precision

Accuracy and precision (intra- and inter-day) were calculated from three different standard curves on three separate days. Six replicates of QC samples at four different concentrations (LLOQ, LQC, MQC, and HQC) were analyzed. Precision was defined as the percent relative standard deviation (%RSD) and accuracy as the percent bias (% bias). To be within the acceptance criteria, accuracy and precision were required to be within ± 15% for each concentration, except for the LLOQ, where ± 20% was acceptable. The results of the calibration curve and QC samples were evaluated and documented prior to the start of each batch according to the FDA guidance^34^.

#### Absolute extraction recovery and matrix effect

Extraction recovery was evaluated in DBS samples by calculating the ratio of the analyte response for pre-spike and post-spike samples multiplied by 100 at three different QC concentrations (LQC ,MQC, HQC). Pre-spike samples were prepared by adding 2.5 μL of working stock to the biological matrix (blood) and processed as per extraction method. Post-spike samples were prepared by processing blank DBS and spiked BDS samples as outlined in Section “Preparation of DBS and extraction method”. For both the pre-spike and post-spike conditions, samples for all analytes were prepared at the LQC, MQC, and HQC levels in triplicate.

The matrix effect was evaluated by calculating the ratio of the analyte response for post-spike and aqueous standards sample multiplied by 100 for each of the three different QCs.

#### Dilution integrity

High concentration dilution integrity QCs with concentrations of 5000 and 10,000 ng/mL were prepared in replicates of six in blood. These QCs underwent two- (2 × HQC), five- (5 × HQC), and ten-fold (10 × HQC) dilutions. The calculated concentration measurements were compared to the nominal concentration at each dilution level, with acceptance criteria for each dilution factor required to be within 15% for accuracy (% bias) and within 15% for precision (% relative standard deviation).

### Stability

Stability of AZI, HCQ, DCQ, BDCQ, DHCQ and IS in DBS samples was determined at various conditions expected to arise during sample handling and storage, the following conditions are: bench-top (20 °C, 50 days), extracted samples in auto-sampler (4 °C, 36 h) and long-term neat stock (–80 ± 5 °C, 8 months). Each stability condition was assessed using (LQC, MQC, and HQC) samples in replicates of six as the stability samples. The samples were analyzed and were deemed stable when accuracy and precision results did not deviate more than ± 15% from the nominal value.

### The blood to plasma ratio (Kb/p)

In a 37 °C water bath, 800 µL of fresh human blood was incubated for 10 min prior to drug spinking. Blood was spiked with the drug at concentrations of 1 µM for all analytes to maintain organic content < 1%. Aliquots of 50 µL were collected at time points (0, 30, 60 min). Additional samples of 120 µL were collected in a micro-centrifuge tube then centrifuged at 4000 × *g* for 10 min at 4 °C to extract 50 µL plasma. For matrix match, blank plasma of 50 µL was added to the collected blood aliquot, and 50 µL of blank blood was added to the extract 50 µL plasma. The samples were further processed as per Section “Preparation of DBS and extraction method”. The analytes' peak area ratio was used to calculate the blood-to-plasma (B/P) ratio using the following equation: blood-to-plasma (B/P) ratio = Concentration _Blood_/ Concentration _Plasma_.

### Study design and blood collection

All developed methods were performed in accordance with the relevant guidelines and regulations as part of two randomized multi-center clinical trials (NCT04328961 and NCT04354428). All the procedures involving handling human DBS samples were approved by the Institutional Biosafety Committee (IBC protocol number 20-04-025-BL1) of the University of Nebraska Medical Center (UNMC). The study was approved by the institutional review board at the clinical sites where the samples were collected. Written informed consent was obtained from all participants. Enrollment was up to 495 eligible adults ages 18–80 years with high risk for lower respiratory tract infection progression at baseline who are PCR-confirmed SARS-CoV-2 infection (165 per arm). Participants in the post-exposure prophylaxis or treatment of COVID-19 study were administered 400 mg HCQ daily for 3 days, followed by 200 mg daily for 11 days (ClinicalTrials.gov; NCT04328961)^31^. For the clinical trial HCQ treatment of COVID-19, SARS-CoV-2( +) subjects were administered HCQ at 400 mg twice daily for 1 day, followed by 200 mg twice daily for the remaining 9 days with and without azithromycin (ClinicalTrials.gov; NCT04354428)^32^. Up to 200 participants were enrolled in the DBS sub-study with the aim to evaluate HCQ and AZI drug concentrations and the exposure–response relationship of HCQ. Whole blood collection was randomized at 1 to 5 times after dosing had commenced. However, no more than 1 sample per day was collected. Whole blood for DBS sampling was obtained using a sterile finger pricker to puncture the tip of the finger. Approximately 100 μL of blood was applied to the DBS filter card. The DBS was left to dry for at least 3 h. All the samples were shipped and handled as biohazardous materials and shipped to the clinical trial site in accordance with the applicable regulations and stored at − 20 °C. All samples were then shipped and handled as biohazardous materials to UNMC, Omaha, NE, USA and stored at − 80 °C until processed for analysis.

## Results and discussion

### Chromatographic and mass spectrometric conditions optimization

**Figure 1:**
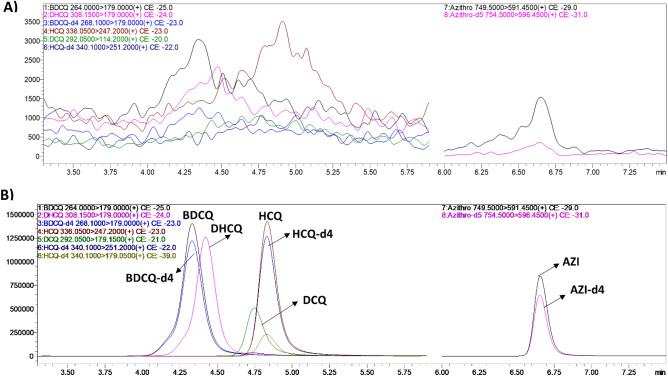
Representative MRM ion-overlay chromatograms (**A**) extracted blank DBS and (**B**) standard spiked at 500 ng/mL extracted from DBS. (*AZI*) Azithromycin, (*HCQ*) hydroxychloroquine, (*DCQ*) desethylchloroquine, (*BDCQ*) bisdesethylchloroquine, (*DHCQ*) monodesethylhydroxychloroquine.

To optimize MS/MS conditions to quantify AZI, HCQ, DCQ, BDCQ, DHCQ, and IS (HCQ-d4, BDCQ-d4, and AZI-d3), both electrospray ionization (ESI) and atmospheric pressure chemical ionization (APCI) conditions were tested. The signal intensity was highest for all analytes utilizing the ESI source in positive mode (data not shown). Fragment specific mass spectrometer parameters were optimized using 1 µg/mL of the analyte. Protonated molecular ions [MH]^+^ of AZI, HCQ, DCQ, BDCQ, DHCQ, HCQ-d4, BDCQ-d4, and AZI-d3 gave a unique precursor ion > product ion combinations, which allowed selective identification and quantification of the analyte without interference from the IS (Table 2). No endogenous interfering peaks were identified at the retention times of the analytes of interest (Fig. 1).

The compound dependent parameters such as voltage potential Q1 (V) and Q3 (V), and collision energy (CE) were optimized to obtain the highest signal intensity for all the analytes and IS (Table 2).

The analytical column was an AQUASIL C18 (5 μm, 50 × 4.6 mm, ThermoScientific, Waltham, MA) protected with a AQUASIL C18 guard column (ThermoScientific, Waltham, MA). The mobile phase consisted of 0.2% FA in water and MeOH acidified with 0.1% FA at a flow rate of 0.5 mL/min and a column oven operated at 40 °C. The mobile phase was operated in gradient mode described in (Table 1). The retention times of 6.6, 4.8, 4.7, 4.3, 4.3, 4. 8, 4.3, and 6.63 min for AZI, HCQ, DCQ, BDCQ, DHCQ, HCQ-d4, BDCQ-d4, and AZI-d3, respectively. Total run time was 8.1 min with an additional 2.8 min were added after the final analyte peak to allow the column to re-equilabrate.

### Hematocrit effect

The accuracy across all concentration levels is within an acceptable ± 15%. This indicates negligible ion suppression due to changes in Hct (Table 3).

**Table 3:** Effect of different Hct values on accuracy of QC samples for AZI, HCQ and its metabolite from human DBS (Mean ± SD, n = 3).

Analyte	Hct effect: accuracy (% nominal, Mean ± SD, n = 3)					
	Hct:0.20		Hct:0.42		Hct:0.60	
	LQC	HQC	LQC	HQC	LQC	HQC
AZI	97.75 ± 14.2	103.15 ± 0.5	107.5 ± 4.1	100.8 ± 2.1	101.9 ± 20.4	98.85 ± 5.2
HCQ	86.6 ± 2.4	91.4 ± 1.3	93.5 ± 8.9	109.7 ± 10.8	88.5 ± 9.8	105.5 ± 5.8
DCQ	88.3 ± 1	92.6 ± 1.9	97.3 ± 7.8	110.8 ± 10.5	94.6 ± 8.7	106.7 ± 6.7
BDCQ	86.2 ± 7.7	91.9 ± 2	92.8 ± 5.1	108.2 ± 10.2	87.7 ± 8.1	103.2 ± 4.7
DHCQ	89.8 ± 1	86.2 ± 2	94.7 ± 9.2	102.1 ± 10.0	92.7 ± 1.5	99.5 ± 3.8

### Assay validation

#### Sensitivity, specificity and selectivity

The sensitivity of the method was determined by the concentration with at least a 10:1 signal to noise ratio. The assay sensitivity varied from 1 to 200 ng/mL for all analytes corresponding to an on column sensitivity ranging from 0.11 to 217.39 pg. The LLOQ had acceptable inter and intra—day accuracy and precision for all analytes (Table 4). The specificity and selectivity were determined by the analyzed blank blood from six different donor, extracted for potential interferences at the retention times of the analytes and IS. There was no significant interference or co-eluting peaks that were > 20% of the LLOQ for each analyte, and no co-eluting peaks > 5% of the area of IS were observed (Fig. 1).

**Table 4:** Assessment of precision (% R.S.D.) and accuracy (% Bias) of HCQ, DCQ, BDCQ, DHCQ, and AZI in DBS.

	LLOQ				LQC				MQC				HQC			
	% RSD		% Bias		% RSD		% Bias		% RSD		% Bias		% RSD		% Bias	
	Inter-day	Intra-day	Inter-day	Intra-day	Inter-day	Intra-day	Inter-day	Intra-day	Inter-day	Intra-day	Inter-day	Intra-day	Inter-day	Intra-day	Inter-day	Intra-day
HCQ	7.5	3.1	7.8	8.7	7.4	5.5	11.3	− 0.2	6.2	5.1	3.2	− 1.2	7.9	4.5	− 3.2	− 12
DCQ	6.0	8.2	− 11	0.9	3.5	5.7	6.4	8.1	7.3	8.8	5.3	7.6	7.1	4.3	3.6	− 3.6
BDCQ	4.5	11.1	− 5.8	4.5	7.1	8.1	− 7.7	− 5.1	8.6	4.1	− 0.3	8.4	5.1	7.9	− 5.3	− 6.9
DHCQ	11.9	11.7	− 3.9	3.1	8.2	8.8	2.5	− 0.6	5.7	3.4	− 0.2	− 0.9	7.7	5.2	2.1	5.9
AZI	13.57	8.56	− 11	− 6.4	8.6	5.8	− 4.7	5.3	5.6	3.3	− 6.9	− 1.9	9.14	0.8	11.6	6.5

#### Accuracy and precision

Intra- and inter-day accuracy and precision for AZI, HCQ, DCQ, BDCQ, and DHCQ were assessed at four different concentrations (LLOQ, LQC, MQC, and HQC, n = 6) from three different validation on three separate days. Intra- and inter-day The precision (% RSD) and accuracy (% Bias) results are shown in Tables 4. The Precision (% RSD) ranged from 0.84 to 13.57% for all analytes. Accuracy (%Bias) ranged − 12.3 to 11.3%, within the acceptable limits at all concentration levels indicating acceptable assay accuracy. Intra- and inter-day accuracy and precision of the all analytes was within the acceptance limits at all concentration levels.

#### Calibration curve and linearity

**Figure 2:**
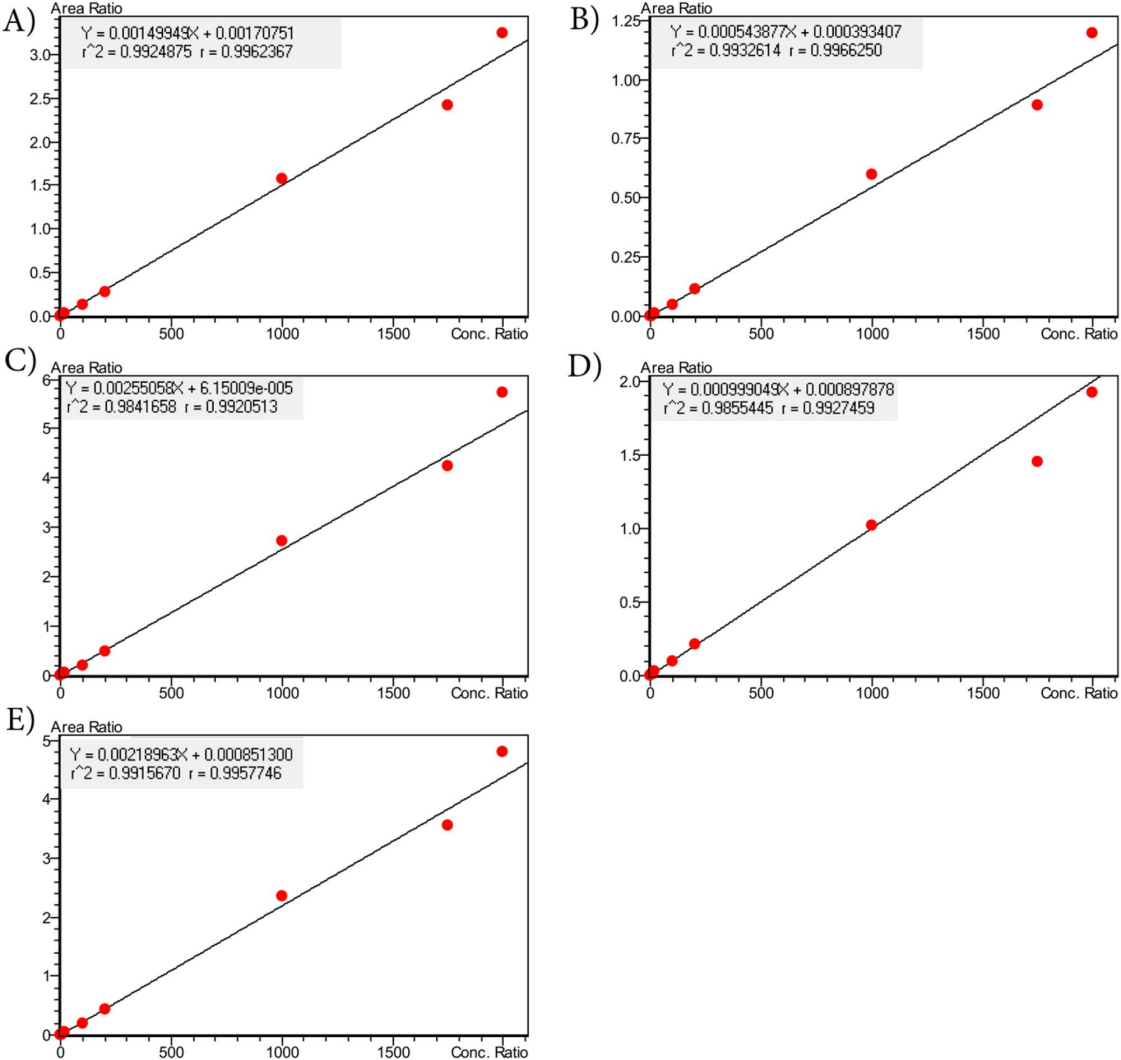
Linear response over concentration range of 1 to 2000 ng/mL for (**A**) HCQ, (**B**) DCQ, (**C**) BDCQ, (**D**) DHCQ, and (**E**) AZI.

The method displayed a linear response over the concentration range from 1 to 2000 ng/mL or on column sensitivity ranges 0.11 to 217.39 pg for all analytes, with a correlation coefficient (r^2^) of 0.98 or better (Fig. 2). The data were fitted using linear regression with the use of 1/*x*^2^ weighting. The lowest concentration with RSD < 20% was taken as LLOQ and was found 1.0 ng/mL.

#### System suitability and carry-over

Prior to each validation, five system suitability samples (HQC, 1500 ng/mL) were injected into the LC–MS/MS and the %CV was ≤ 6. Analytes and IS peak showed minimal variability in retention time with an RSD well within the acceptable limit of ± 5%. To prevent carryover, a needle wash was included after every injection with MeOH/H_2_O (9:1) spiked with 0.1% FA. The analytes showed no significant peak (< 20% of the LLOQ) in any samples injected after the HQC samples. No co-eluting peaks > 5% of the area of IS were observed. Thus, there was no significant carry over effect.

#### Absolute extraction recovery and matrix effect

The extraction methods of AZI, HCQ, and its metabolites from DBS samples were stepwise evaluated by comparing the analyte (LQC—3 ng/mL, MQC—500 ng/mL, and HQC—1500 ng/mL) response of spiked pre-extraction and post-extraction samples by testing the absolute extraction recoveries (AER) and absolute matrix effects (ME) (Table 5). The phospholipid removal Phree 96 plate was used for better clean-up and phospholipid removal with high analytes and IS AER > 85% from DBS Samples (Table 5). The AER and ME were consistent throughout the calibration curve. Therefore the validation could be performed with an LLOQ of all analytes of 1 ng/mL, corresponding to on column sensitivity of 0.11 pg. The same extraction method was applied for the extraction of 25 µL plasma and blood samples with AER > 90% (Data not shown). The sample clean-up method was efficient and reproducible as shown by the results of the method validation. All samples utilized two (3 mm each) DBS punches, equivalent to ~ 10 µL of blood, for sample preparation. A total of 10 µL of the finale-extracted solution was injected to LC–MS/MS. The method of extraction was highly efficient regardless of the final solution being highly diluted. Optimum selectivity and sensitivity were achieved with 0.11 pg on column. The AER and ME are shown in (Table 5).

**Table 5:** Absolute extraction recoveries and absolute matrix effects for HCQ, DCQ, BDCQ, DHCQ, and AZI in DBS. (Mean ± SD, n = 5).

Analytes	% absolute extraction recoveries (Mean ± SD, n = 3)			% matrix effect (Mean ± SD, n = 3)		
	LQC	MQC	HQC	LQC	MQC	HQC
HCQ	98.8 ± 1.9	94.1 ± 6	102.2 ± 1.2	98.8 ± 0.6	94.1 ± 3.1	102.2 ± 0.9
DCQ	88.5 ± 6.2	88 ± 4.4	97.4 ± 3.1	88.5 ± 6.3	88 ± 0.5	97.4 ± 2.7
BDCQ	87.8 ± 1.5	91.3 ± 6	100.1 ± 0.9	87.8 ± 4.8	91.3 ± 2.9	100.1 ± 0.6
DHCQ	91.7 ± 2.3	93.8 ± 6.5	102.9 ± 2	91.7 ± 1.6	93.8 ± 2.4	102.9 ± 1.7
AZI	91.9 ± 3.3	114 ± 0.21	86.9 ± 0.29	97.7 ± 4.4	93.4 ± 0.31	119.6 ± 1.2
HCQ-d4	96.6 ± 3.8			101.2 ± 1.7		
BDCQ-d4	100 ± 15			99.31 ± 0.23		
AZI-d3	98.2 ± 5.2			92.15 ± 1.3		

### Dilution integrity

Dilution of QC samples above the upper limit of quantification (ULOQ) with like matrix to bring the concentration to within the quantitation range 1.0–2000 ng/mL was performed for three concentrations. The mean analyzed value of all diluted samples was within 15% of nominal concentration, with precision of the replicates ± 15% coefficient of variation and the precision was within 15% RSD for each of the dilution factors (data not shown), indicating study samples could be diluted up to tenfold with acceptable precision.

### Stability

The stability of AZI, HCQ, and it’s metabolites were evaluated in human DBS samples, where the mean concentration was expressed as percentage accuracy from nominal concentration. All analytes and metabolites were found to be stable and within ± 15% of the actual concentration at bench-top (20 °C, 50 days), auto-sampler (4 °C, 36 h), and long-term neat stock (– 80 ± 5 °C, 8 months). Data are presented in (Table 6).

**Table 6:** Mean stability for HCQ, DCQ, BDCQ, DHCQ, and AZI in DBS.

Analyte	Accuracy (% nominal, mean ± SD, n = 3)					
	DBS at Bench-top (50 days at 20 °C)		Auto-sampler (4 °C, 36 h)		Long-term neat stock (8 months at –80 ± 5 °C)	
	LQC	HQC	LQC	HQC	LQC	HQC
HCQ	110.3 ± 1.6	90.1 ± 0.6	96.5 ± 9.1	96.8 ± 16.9	110.7 ± 3.3	93.1 ± 1.2
DCQ	110.1 ± 6.9	90.3 ± 0.8	107.9 ± 1.7	99.4 ± 1.6	103.95 ± 1.9	96.3 ± 3.8
BDCQ	116.8 ± 1.0	91.1 ± 0.7	87.95 ± 4.7	94.2 ± 0.9	84.55 ± 1.9	102.3 ± 1.5
DHCQ	110.0 ± 2.1	93.1 ± 0.6	99.35 ± 1.6	89.5 ± 3.0	108.6 ± 0.4	99.1 ± 6.5
AZI	118.1 ± 0.8	86.9 ± 0.4	94.15 ± 0.9	92.7 ± 11.9	100.1 ± 7.5	89.2 ± 0.4

### The blood to plasma ratio (Kb/p)

The blood:plasma (Kb/p) ratio was determined at a concentrations of 1 µM after 0, 30- and 60- min incubations at 37 °C. (Table 7). Higher blood:plasma (Kb/p) ratio indicates that a larger proportion of the drug is binding to blood components rather than remaining in the plasma. It also impacts the interpretation of drug concentration measurements in plasma and may not accurately reflect the total drug exposure in the body.

**Table 7:** Blood to plasma ratio (B/P) of HCQ, DCQ, BDCQ, DHCQ, and AZI in DBS.

Time (minutes)	Blood to plasma ratio (Mean, n = 3)				
	AZI	HCQ	DCQ	BDCQ	DHCQ
0	1.06	1.7	3.2	2.0	1.8
30	1.36	1.9	4.2	3.0	2.2
60	1.43	1.9	3.5	2.9	1.8

### Clinical application of the method for pharmacokinetic studies and significance

The method was successfully applied to the quantification of AZI, HCQ, and metabolite concentrations for more than 2000 DBS samples. The DBS sample concentrations for AZI and HCQ ranged from 1.1 to 141.1 ng/mL and 0.8–24,941.1 ng/mL, respectively. The developed method was utilized to evaluate variability in drug concentrations following prescribed fixed dosing and build pharmacokinetic models to help understand the drug ADME properties in humans. The physiologically based pharmacokinetic model for HCQ and metabolites for this study have recently been reported^35^. The use of DBS sample collection during a pandemic allowed the rapid collection of samples from remote locations utilizing minimal storage requirements, excellent sample stability, and ease of sample processing. The use of DBS sampling has received widespread applications in bioanalytical and forensic toxicology in the past few years. DBS offers advantages, including the use of a small sample requirement (*e.g.,* 10 µL), which facilitates sampling in special populations like infants and younger children where conventional blood collection is difficult. DBS also provides convenience to the patient as it is a less invasive sample collection compared to other techniques, has low biohazard risk, and easy shipment of the DBS filter card^27,28,36,37^. DBS can also improve diversity among clinical trial participants located in remote or low-resource settings where access to phlebotomy and refrigeration for specimen transport are limited, or among populations unable to leave home due to personal health or population health circumstances, including stay-at-home orders during a pandemic^38^. However, when using DBS for bioanalytical sample collection, it is important to consider certain factors during sample collection and handling to ensure accurate results. The DBS analytical performance can vary depending on the absorbent filter paper used for sample collection and blood spotting technique. Ideally, only one blood drop should be collected per DBS sample, and care should be taken to avoid hemolysis of the specimen, which can result from touching the filter paper and excessive squeezing of the puncture site. Hemolysis can affect the results. However, this can be overcome by patient training and recommending the patient to fill at least three complete circles with blood^37^. Another predominant source of analysis variability in DBS is Hct. Blood samples with an elevated Hct may have increased viscosity and result in a less homogenous sample spread on the filter paper compared to a sample with lower Hct^37^. We evaluated the impact of Hct on accuracy and precision and found Hct to not impact our results. In addition, we used two punches from the filter paper for sample extraction. Abnormal lipids in blood samples, known as lipemia, is another crucial factor to consider while conducting sample analysis^39^. The interference caused by lipemia can be overcome by implementing an extraction method to remove both triglycerides and phospholipids. The degree of interference, however, depends on the type of assay^40^. We observed no ion suppression in our sample analysis.

Finally, DBS can reduce research costs such as phlebotomy and transportation, self-collected decreasing the burden on clinical sites, which can be significant, especially with large cohorts as in this study^27,28,36^.

### Methods limitation

While our method demonstrates robustness and provides accurate results for DBS analysis, certain limitations should be acknowledged. First, it is important to note that selectivity and specificity testing specifically for hemolytic and lipemic whole blood samples was not performed as part of this study. Hemolysis and lipemia can potentially interfere with the analysis and affect the accuracy of results. Additionally, it is important to recognize that the method lacks specificity testing of analytes in the presence of co-administered medications. The potential interaction between analytes and co-administered medications may influence the accuracy and specificity of the results.

While our method addresses certain limitations, such as Hct impact, multiple punch extraction, and lipemic interference, further studies are warranted to evaluate selectivity and specificity in hemolytic and lipemic samples, as well as to assess the specificity of analytes in the presence of co-administered medications. These limitations are crucial for a comprehensive understanding of the method's performance and its applicability in different clinical scenarios. Further studies addressing these limitations would provide valuable insights and enhance the robustness and applicability of the DBS analysis method.

## Conclusion

We developed and validated a robust, sensitive, and reproducible LC–MS/MS method for quantitate AZI, HCQ, and its metabolites (DCQ, BDCQ, and DHCQ) in human blood utilizing a DBS sample collection technique. The DBS sampling method was advantageous with simple sample collection, a limited amount of blood sample required, easy transfer and storage conditions, and exhibited excellent drug stability. The LC–MS/MS bioanalytical method was validated according to FDA guidelines. The method showed superior selectivity and sensitivity, with nominal concentrations as low as 1 ng/mL or 0.11 pg on column, compared to other developed methods with LLOD of 25 ng/mL. The method showed no significant matrix effects and acceptable accuracy and precision that fulfilled the FDA requirements. The current LC–MS/MS method provides a valuable tool in evaluating the pharmacokinetic/pharmacodynamic properties of AZI, HCQ, and its metabolites as a single agent or in combination with other drugs.

## Data Availability

The datasets generated and analysed during the current study are not publicly available to comply with HIPPA accountability act but are available from the corresponding authors on reasonable request.
